# The Organon in Our Colleges

**Published:** 1885-03

**Authors:** M. D. Lummis

**Affiliations:** Los Angeles, Cal.


### THE ORGANON IN OUR COLLEGES.

Editor "Homœopathic Physician":—In an article in the Homœopathic Physician of November, 1884, Dr. P. P. Wells, in replying to Dr. McKibben in regard to the teachings of our medical colleges, makes this rash remark: "We receive with great pleasure and thankfulness the statement that there is *one* college in the country where the *Organon* is taught, and that this is in St. Louis. Is there another? If there be, its whereabouts is unknown." I cannot but think that Dr. Wells is in a similar situation to the old New Hampshire deacon, who appropriated a raft and all its supplies, and when asked if he could imagine where it came from, replied solemnly, "Bretherin, I never knowed!" Has Dr. Wells inquired of those who know as to the teaching or non-teaching of the *Organon?* If so, he surely would have found out that in the Boston University School of Medicine, the *Organon* is not only taught, and taught well, but that the undergraduates are "quizzed" upon its teachings, thus giving them a double chance to learn its strength and weakness, and to ventilate their own views and have them rectified or accepted. I hope this information will add to the "pleasure and thankfulness" of not only Dr. Wells, but of all good homœopathists.

M. D. Lummis, M. D.

Los Angeles, Cal.

